# The Visual Acuity Outcome and Relevant Factors Affecting Visual Improvement in Pediatric Sporadic Chiasmatic–Hypothalamic Glioma Patients Who Received Surgery

**DOI:** 10.3389/fneur.2020.00766

**Published:** 2020-08-19

**Authors:** Chihyi Liao, Heng Zhang, Zhiming Liu, Zhe Han, Chunde Li, Jian Gong, Wei Liu, Zhenyu Ma, Yongji Tian

**Affiliations:** ^1^Department of Neurosurgery, Beijing Tiantan Hospital, Capital Medical University, Beijing, China; ^2^Beijing Key Laboratory of Brain Tumor, China National Clinical Research Center for Neurological Diseases, Center of Brain Tumor, Beijing Institute for Brain Disorders, Beijing, China

**Keywords:** chiasmatic–hypothalamic glioma, optic pathway glioma, visual acuity, prognostic factors, primary surgical treatment

## Abstract

**Background:** The role and effectiveness of primary surgical treatment for sporadic chiasmatic–hypothalamic glioma (CHG) are not clear. The present study was to describe sporadic CHG visual acuity (VA) outcomes after surgery and to analyze the relevant factors affecting VA improvement.

**Methods:** Forty-five pediatric sporadic CHG patients who met the inclusion criteria were included in a retrospective study. All patients received primary intratumor partial resection. Disease characteristics, treatment strategies, complications, and VA outcome were analyzed. Univariate and multivariate analyses were performed to identify relevant factors of VA improvement. Receiver operating characteristic (ROC) analysis was performed to evaluate the predictive accuracy of measurement indexes.

**Results:** There were 77 eyes of 45 children suffering from various levels of VA impairment before surgical treatment, and only 13 eyes had normal vision. Patients with resection extents >70, 50–70, and <50% accounted for 26.67, 24.44, and 48.89%, respectively. The percentages of VA maintained and deteriorated in normal vision eyes were 61.54 and 38.46%. The percentages of VA improved, maintained, and deteriorated in visually impaired eyes after surgery were 29.87, 45.45, and 24.68%, respectively. There was a positive correlation between the IVA level and VA improvement. There was no significant difference in VA improvement between the different resection extents. Blindness occurred in ~4.4%. Approximately 11.1% of the children had complications that affected quality of life, which correlated with resection extent. IVA and tumor size were correlated with VA improvement. The AUC for IVA + tumor size predicting VA improvement was 0.831. The cutoff points for IVA level and tumor volume were 4.5 and 43.50 cm^3^, respectively.

**Conclusions:** IVA and tumor size were correlated with VA improvement after primary intratumor partial resection. Children with IVA ≥ level 5 were more likely to achieve visual improvement after decompression surgery, but decompression had limited effectiveness on vision improvement in patients with tumor volumes ≥ 43.50 cm^3^. Performing resections < 50% was safe and did not reduce the effect of decompression to improve VA.

## Introduction

Optic pathway glioma (OPG) is a multidisciplinary and neoplastic disease that originates from any part of the visual pathway or hypothalamus (1). The natural history of OPG is unpredictable, and it can present as symptomatic or asymptomatic (2). OPG accounts for ~4–6% of all pediatric intracranial tumors, and it most frequently presents as pilocytic astrocytoma and pilomyxoid astrocytoma but may also present as high-grade malignant glioma (3). OPG is classified as neurofibromatosis type 1 (NF-1) or sporadic, and it may be anatomically distinguished using the Dodge classification and other modified classification methods (4, 5). Sporadic OPG, particularly chiasmatic–hypothalamic glioma (CHG), may be more aggressive than NF-1 (6, 7). The tendency of CHG to occur in children adds to the complexity of disease management (7). Chemotherapy is widely used as the first-line treatment for OPG, and surgery and radiotherapy are used more often in severe or recurrent cases. The role of surgical treatment of the disease is to preserve VA, relieve intracranial pressure, reduce tumor burden, and control intractable progression (8, 9). Although primary surgery showed good efficacy and safety for CHG patients in some studies (10, 11), information on the effectiveness of surgery to improve visual acuity (VA) is limited. The identity of patients who are more likely to benefit from surgery, and the extent that is low risk and effective for decompression are not known. The present study described sporadic CHG VA outcomes after surgery and analyzed the relevant factors affecting VA improvement.

## Methods

We identified all children with sporadic CHG who received primary surgical treatment between 2012 and 2018 from Beijing Tiantan hospital databases. All of the patients received intratumor partial resection to achieve decompression and cytoreduction. Indications for patients to receive surgery included (1) exophytic tumor mass causing progression of neurological deficit or visual impairment, (2) large-size tumor causing a space occupying effect, and (3) tumor involving the third ventricle and causing hydrocephalus. Patients were included in this study (1) if they received VA assessment in the Beijing Tiantan Hospital Ophthalmology Department, (2) if they received intratumor partial resection as primary treatment, (3) if they had complete IVA data, and (4) if their last visual acuity (LVA) was assessed at least 1 year after surgery. Initial visual acuity (IVA) was assessed within 1 week before surgery. Patients were excluded if they had any other ophthalmic diseases or if they had not finished AT when LVA was assessed. Age, sex, clinical manifestation, surgical approach, pathology, tumor size, resection extent, adjuvant treatment (AT), IVA and LVA data, and post-surgical complications were recorded for each patient.

**Figure 1:**
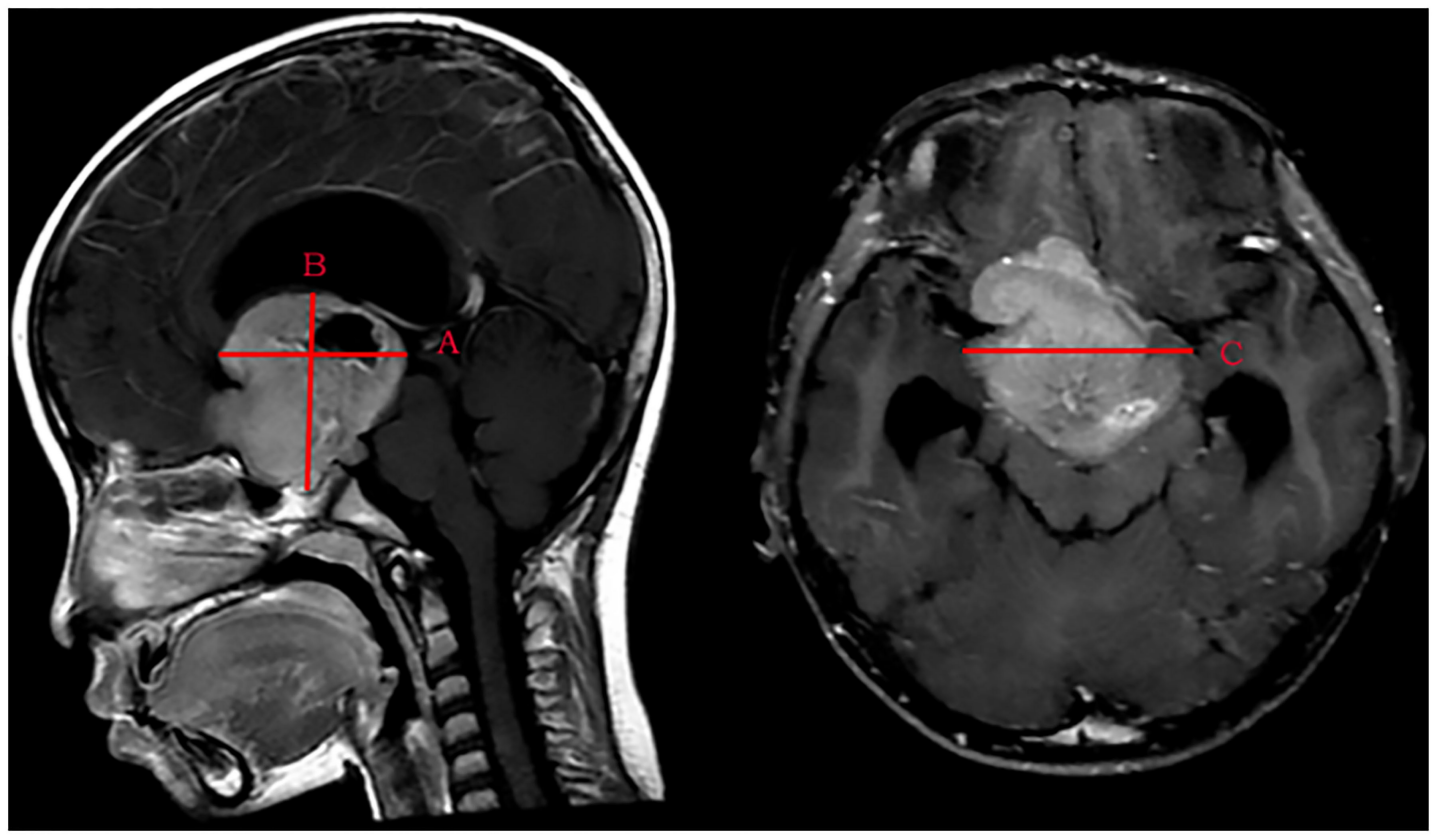
Measurement of the maximum distance on three standard planes of MRI images. (A) The maximum anteroposterior distance of the tumor is parallel to the AC-PC-line. (B) The maximum craniocaudal distance of the tumor is perpendicular to the axial plane. (C) The maximum transverse distance of the tumor is perpendicular to the sagittal plane.

According to the range of vision loss reported by the International Council of Ophthalmology (12), we converted the categories of vision impairment to a seven-level scale: level 7 to level 1 represent normal vision (≥0.8), mild visual impairment (0.32–0.63), moderate visual impairment (0.125–0.25), severe visual impairment (0.05–0.1), profound visual impairment (0.02–0.04), near-blindness (<0.02), and light perception or blindness, respectively. The VA outcome of patients was categorized into “VA improvement” and “no VA improvement.” If the LVA level was higher than the IVA level, the outcome was defined as “VA improvement.” If not, the outcome was defined as “no VA improvement.” “Maintained” was used when the LVA level was equal to the IVA level, and “deteriorated” was used when the LVA level was lower than the IVA level. Tumor volume was calculated using the ellipsoid volume formula “A × B × C × π/6” (Figure 1) (13). Tumors were divided into small, medium, and large size groups by tertiles. Tumor volume ≤13.80 cm^3^ was categorized as a small-size tumor, 13.80 cm^3^ < tumor volume ≤34.60 cm^3^ was categorized as a medium-size tumor, and the tumor volume > 34.60 cm^3^ was categorized as a large-size tumor.

Continuous data are presented as medians (25th percentile, 75th percentile) or means ± standard deviation according to non-normal or normal distributions, respectively. Categorical data are presented as numbers (percentage). Correlations between the VA outcome and IVA were assessed using the gamma test. Chi-squared test was used for comparisons between groups, and Fisher's exact test was used when appropriate. With the interconnection of two eyes in single patient considered, multivariate analysis was performed using logistic regression within generalized estimating equations (GEE). To avoid the omission of prognostic factors, factors with *p* < 0.1 in the univariate analysis were entered into the multivariate analysis. With the area under the curve (AUC) and the Youden index computed, receiver operating characteristic (ROC) analysis was performed to evaluate the predictive accuracy and identify the cutoff point. Data analyses were performed using IBM SPSS Statistics 20. Two-tailed *p* < 0.05 was considered statistically significant.

The Ethics Review Committee of Beijing Tiantan Hospital approved the study before the investigation began.

## Results

Fifty-eight sporadic CHG patients who received primary intratumor partial resection had IVA assessed before surgery. Forty-five of these patients who had finished AT and had their LVA assessed at least 1 year after surgery were included in this study. The most common clinical manifestation of the included patients was vision impairment (97.77%), and ~55.56% of patients presented with intracranial hypertension (ICH). There were 26.67% patients who received shunts due to the consciousness disturbances caused by ICH. Patients with resection extents >70, 50–70, and <50% accounted for 26.67, 24.44, and 48.89%, respectively. Fourteen children (31.11%) had tumors that were pathology diagnosed as pilocytic astrocytoma (PA), and 20 children (44.44%) were diagnosed with pilomyxoid astrocytoma (PMXA) or pilocytic astrocytoma with pilomyxoid features (PA/PMXA). Eight children (17.78%) were diagnosed with diffuse astrocytoma, two children (4.44%) were diagnosed with ganglioglioma, and one child (2.22%) was diagnosed with anaplastic astrocytoma. Sixty-four percent of the patients received radiotherapy as further AT after surgery, 6.67% received chemotherapy, 22.22% received radiotherapy and oral temozolomide, and 6.67% received observation. The median time of patients to assess LVA was 33 (19.5, 47.5) months after surgery. There was no significant difference for IVA level between the different tumor size groups (*p* = 0.091), and there was no significant difference in the resection extent between the different tumor size groups (*p* = 0.884). The baseline characteristics of CHG patients are summarized in Table 1.

**Table 1:** Baseline characteristics of 45 sporadic CHG children (per subject).

**Characteristics**	**Median (P25, P75)**	***n* (%)**
Age (years)	6 (5.00, 10.50)	
Tumor volume (cm^3^)	26.02 (10.72, 43.50)	
IVA (level)	4 (2, 6)	
LVA (level)	4 (2, 7)	
Gender		
Male		28 (62.22)
Female		17 (37.78)
Symptom		
Bilateral vision impaired		33 (73.33)
Unilateral visual impaired		11 (24.44)
Nystagmus		5 (11.11)
Strabismus		3 (6.67)
ICH		25 (55.56)
Precocious puberty		9 (20.00)
Hemiparesis		2 (4.44)
Shunt		12 (26.67)
Surgical approach		
Longitudinal fissure approach		21 (46.67)
Transcallosal interforniceal approach		20 (44.44)
Subtemporal approach		4 (8.89)
Resection extent		
>70%		12 (26.67)
50–70%		11 (24.44)
<50%		22 (48.89)
AT		
RT		29 (64.44)
CT		3 (6.67)
RT + Oral temozolomide		10 (22.22)
Observation		3 (6.67)
Pathology		
PA		14 (31.11)
PXA or PA/PMXA		20 (44.44)
Diffuse astrocytoma		8 (17.78)
Ganglioglioma		2 (4.44)
Anaplastic astrocytoma		1 (2.22)
Tumor recurrence		1 (2.22)

*IVA, initial visual acuity; LVA, last visual acuity; ICH, intracranial hypertension; AT, adjuvant treatment; RT, radiotherapy; CT, chemotherapy; PA, pilocytic astrocytoma; PMXA, pilomyxoid astrocytoma*.

**Figure 2:**
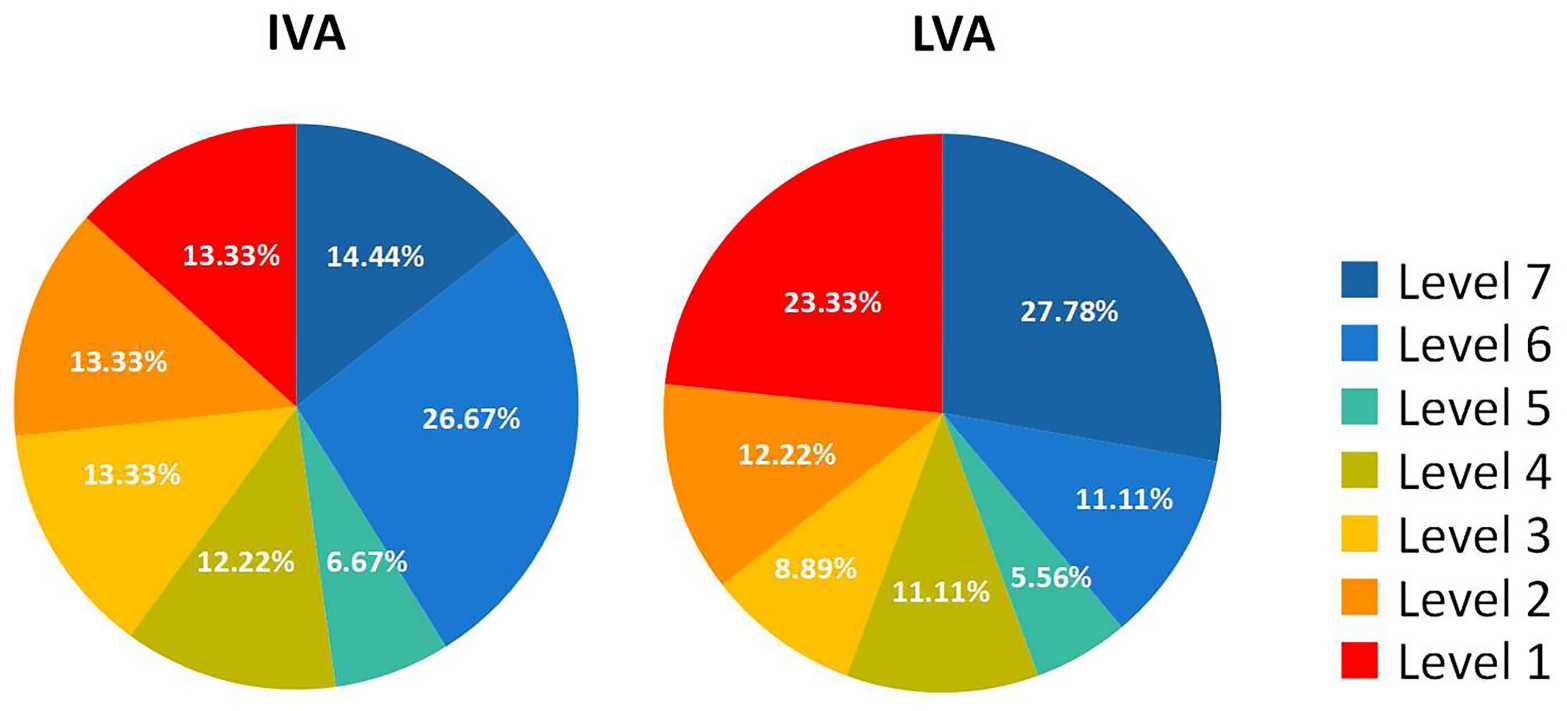
Vision level of IVA and LVA of all sporadic CHG children (per eye).

**Figure 3:**
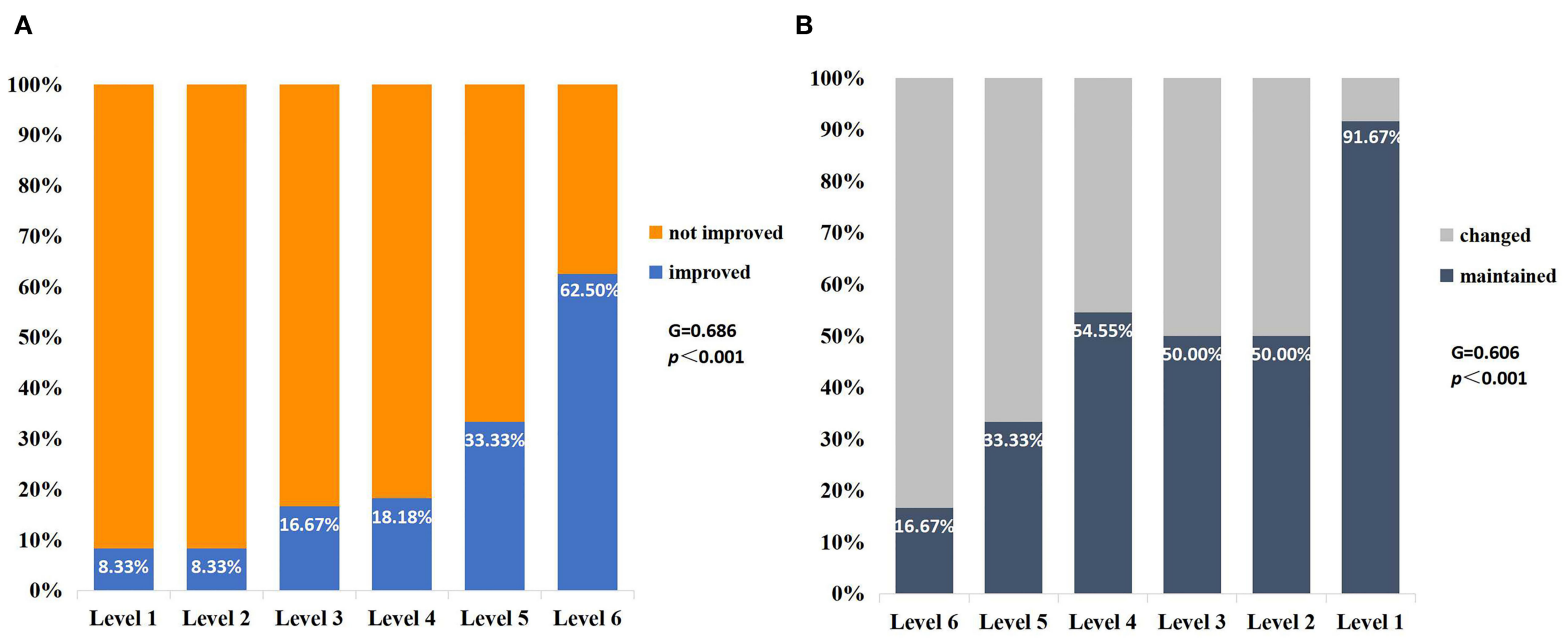
VA outcomes for different vision levels of IVA in the impaired-vision group (per eye). **(A)** There was a positive correlation between the vision level of IVA and VA improvement (*G* = 0.686, *p* < 0.001). **(B)** The number of patients maintaining preoperative VA increased with the decrease in IVA level (*G* = 0.606, *p* < 0.001).

There were 77 eyes of 45 children suffering from various levels of VA impairment before surgery, and only 13 eyes had normal vision. The IVA and LVA data of all eyes are shown in Figure 2 and Table S1 of the Supplementary Material. The percentages of VA maintained and deteriorated in normal vision eyes was 61.54 and 38.46%, respectively (vision dropped to level 6 in four eyes and to level 2 in one eye). The percentages of VA improved, maintained, and deteriorated in vision-impaired eyes after surgery were 29.87, 45.45, and 24.68%, respectively. Two-thirds of patients with VA outcome categorized as “maintained” had IVA equal to or worse than severe visual impairment. There was a positive correlation between the IVA level and VA improvement in vision-impaired eyes (*G* = 0.686, *p* < 0.001). For eyes with lower IVA level, the visual outcomes were more likely to present as “maintained” (*G* = 0.606, *p* < 0.001) (Figure 3).

**Figure 4:**
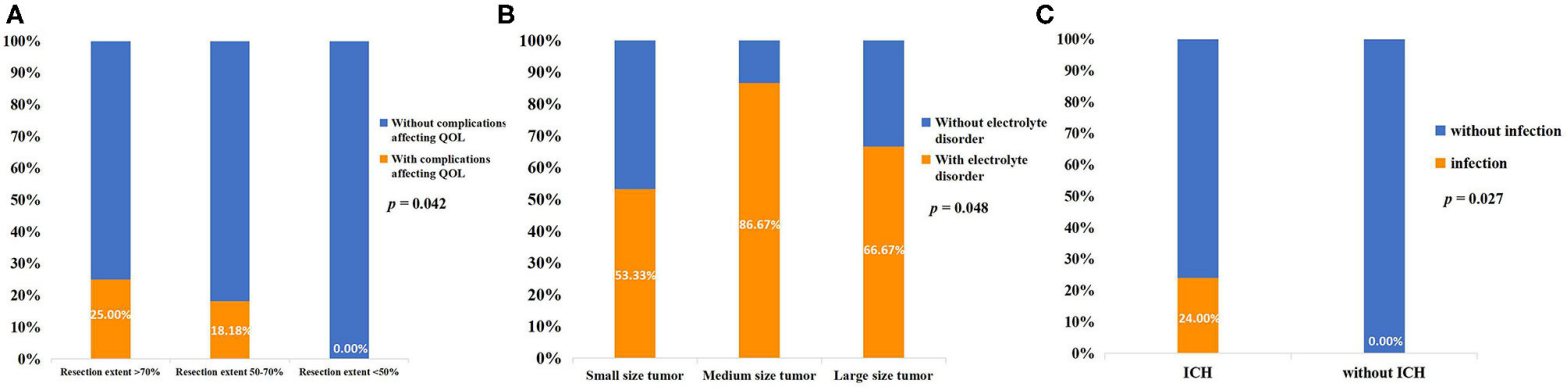
**(A)** Correlation between resection extent and complications affecting the QOL. **(B)** Correlation between tumor size and post-surgical electrolyte disorder. **(C)** Correlation between ICH and post-surgical intracranial infection.

The mean length of hospitalization of the included patients was 15.3 ± 4.2 days. Post-surgical electrolyte disorder occurred in 75.5% of all patients, and the median duration of electrolyte disorder of these patients was 5.5 (3, 8.25) days. Patients with endocrine disorders after surgery accounted for 68.8%, but only three of these patients had long-term endocrine dysfunction that required medication. There were 44.4% of patients with diabetes insipidus, but none of these patients needed long-term medication. Infection occurred in six patients, and subdural effusion occurred in two patients. The complications affecting the quality of life (QOL) were unilateral blindness in two patients and long-term endocrine dysfunction in three patients (Table 2). The two patients who suffered from post-operative unilateral blindness were in the small-size tumor group. The correlations of post-surgical complications with disease characteristics and treatment are shown in Table S2 of the Supplementary Material. There was a correlation between complications affecting QOL and the extent of resection (*p* = 0.042). There was also a correlation between electrolyte disorder and tumor size (*p* = 0.048) and ICH and infection (*p* = 0.027) (Figure 4).

**Table 2:** Post-surgical complications and hospital stay of 45 sporadic CHG children.

**Variable**	**Mean ± Std. Deviation**	**Median** **(P25, P75)**	**No. of eyes (%)**	**No. of patients (%)**
Hospital stay	15.3 ± 4.2			
Complications affecting QOL				5 (11.1%)
Blindness			2 (2.2)	2 (4.4)
Long-term endocrine dysfunction				3 (6.7%)
Endocrine disorder				31 (68.8)
Infection				6 (13.3)
Diabetes insipidus				20 (44.4)
Electrolyte disturbance				34 (75.5)
Duration of electrolyte disorder		5.5 (3, 8.25)		
Subdural effusion				2 (4.4)

*QOL, quality of life*.

Only one 5-year-old female child, whose tumor pathology was PA, suffered radiographic progression in the seventh month after AT (RT + oral temozolomide). Her IVA was level 7 for the left eye and level 1 (light perception) for the right eye. She also had symptoms of precocious puberty after the tumor progression, and her parents perceived that she had obvious VA deterioration. Her last LVA was level 2 for the left eye and level 1 (blindness) for the right eye.

Univariate analysis of the vision-impaired eyes revealed that IVA (*p* = 0.002) and tumor size (*p* = 0.020) correlated with VA improvement, and the correlation between improved vision and ICH was close to statistical significance (*p* = 0.084). There was no correlation between VA improvement and the extent of resection (*p* = 0.592), pathology (*p* = 0.791), AT (*p* = 0.913), or surgical approach (*p* = 0.149) (Supplementary Material, Table S3) Multivariate analysis revealed that VA improvement correlated with IVA (OR, 1.987; 95% CI, 1.249–3.162; *p* = 0.004), and there was a significant difference in VA improvement between medium- and large-size tumors (OR, 0.205; 95% CI, 0.125–2.073; *p* = 0.048). There was a marginal correlation between ICH and VA improvement (OR, 3.280; 95% CI, 0.984–10.931; *p* = 0.053) (Table 3).

**Table 3:** Multivariate analysis of relevant factors related to VA improvement in vision impaired eyes of sporadic CHG (per eye).

**Variable**	**OR**	**95% CI**	***P-*value^a^**
IVA	1.987	1.249–3.162	0.004
ICH	3.280	0.984–10.931	0.053
Tumor size			
Large	0.205	0.125–2.073	0.048
Medium	1		
Small	0.509	0.042–0.987	0.346

a *the p-value was carried out by logistic regression with generalized estimating equations model*.

In the ROC analysis, the AUC of IVA predicting VA improvement was 0.787 (95% CI: 0.674–0.900, *p* < 0.001), and the AUC of IVA + tumor size predicting VA improvement was 0.831 (95% CI: 0.729–0.933, *p* < 0.001). For medium to large tumors, the AUC of tumor volume predicting VA outcome was 0.748 (95% CI: 0.641–0.883, *p* =0.005). The cutoff point of IVA level was 4.5 (sensitivity = 73.91%, specificity = 75.93%, positive predictive value = 56.67%, negative predictive value = 87.23%, accuracy=75.32). The cutoff point of tumor volume was 43.50 cm^3^ (sensitivity = 95.65%, specificity = 31.48%, positive predictive value = 37.29%, negative predictive value =94.44%, accuracy =50.65%) (Table 4).

**Table 4:** The AUC of different measurement indexes.

**Measurement Indexes**	**AUC**	**95s CI**	***P-*value**	**Cutoff point**
IVA	0.787	(0.674–0.900)	<0.01	4.5
Tumor size				
Medium–Large	0.748^*^	(0.641–0.883)	0.005	43.50
IVA + Tumor size	0.831	(0.729–0.933)	<0.01	–

* *the AUC was calculated by shifting the state variable as “not improved”*.

## Discussion

Current treatments for progressive symptomatic OPG include chemotherapy, radiation, and surgery for select patients (8). The aim of treatments for OPG increasingly focuses on improving the patient's QOL, such as VA rescue, but information on OPG long-term VA outcome is scant (13, 14). The largest study of long-term VA outcomes of OPG was reported by Rakotonjanahary et al. (15). Wan et al. (16) also described sporadic OPG VA outcome and the relevant risk factors. Due to the lack of initial VA data, neither report mentioned the trends in visual changes after treatment. Fisher et al. (17) reported the VA prognosis of OPG after treatment, but this report purely focused on NF-1-associated OPG. All of the patients in these studies received chemotherapy as the first-line treatment. To our knowledge, the present report, which includes 45 sporadic CHG patients who received primary intratumor partial resection, is one of the largest studies focusing on VA outcomes.

Compared to other CNS tumors, OPG has a better survival prognosis, but the overall VA outcome of OPG remains unsatisfactory. More than 50% of sporadic OPG patients have severe vision loss in at least one eye (16). Therefore, improving vision is one of the most important challenges for OPG treatment strategies. Chemotherapy deferred radiation without compromising overall survival (18), but a meta-analysis study suggested that chemotherapy did not improve VA outcomes in most children with OPG, and only 14% of patients experienced VA improvement after chemotherapy (19). Other studies also suggested that patients receiving chemotherapy tended to develop a decline in vision over time (20, 21). Radiotherapy effectively prevented tumor progression and provided a better visual outcome, with 25–43% of patients experiencing VA improvement after radiotherapy (22–26). Due to the potential of radiotherapy to cause long-term endocrine dysfunction, cognitive impairment and secondary vascular disease, only children aged 5–7 years or older are considered for this treatment (1, 27). Tumor debulking helps relieve mass effects and preserves neural functions (28), and decompression surgery before AT may provide a better VA outcome for some patients (29). However, the improvement rate after surgery in different studies varies widely. Konovalov et al. (30) studied 45 patients with giant OPG, all of whom received surgical treatment, and found that 54% of these patients had VA improvement at the last follow-up. Hidalgo et al. (10) also reported OPG VA outcomes after first- or second-line surgical treatment and found that only 14% of the children had better vision. Our study found that 29.87% of vision-impaired eyes had VA improvement after primary intratumor partial resection. It cannot be assumed that surgical treatment was effective for VA improvement in most OPG patients. Potential risks of the surgery include infection, hemorrhage, optic nerve damage, and hypothalamus damage. Because advantages and disadvantages coexist for each treatment for OPG, an understanding of the post-operative complications of patients and identification of patients who are more likely to gain VA improvement after surgery would help rationalize treatment strategies.

The most common post-surgical complication in this retrospective cohort study was electrolyte disorder, which was seen in 75.5% of patients, and the median duration of electrolyte disorder of these patients was 5.5 (3, 8.25) days. The second most common post-operative complication was endocrine disorder, which was seen in 68.8% of patients, but only three of these patients required long-term medication. Post-surgical diabetes insipidus was seen in 44.4% of patients, but none of them needed long-term medication. The post-surgical infection rate was 13.3% in all patients, and the mean length of hospitalization was 15.3 ± 4.2 days. Five patients developed post-surgical complications that affected QOL. There was a correlation between electrolyte disorder and tumor size (*p* = 0.048). We assumed that small-size tumors have less involvement in the hypothalamus tissue, and the shorter operation time and less disturbance to the hypothalamus during surgery resulted in the lower incidence of electrolyte disorders. Current studies contain little information on electrolyte disturbances after OPG surgery, and further studies are needed to analyze its risk factors to guide the surgery and post-operative care. Antibiotic use, operation time, drainage device, and post-operative cerebrospinal fluid leakage correlated to post-craniotomy infection (31). The presence of a ventriculoperitoneal shunt may also correlate with post-surgical infection (32). However, due to the small number of included patients in the present study and the lack of relevant data, we only found a correlation between ICH and infection (*p* = 0.027). There was also a correlation between complications affecting QOL and the extent of resection (*p* = 0.042), and none of the patients with resection extent <50% developed complications affecting QOL. Because there was no significant difference in VA improvement between the different resection extent groups, the performing of a resection with an extent of 50% was low risk and did not reduce the effect of decompression to improve VA. However, the indication for surgery should be estimated strictly, and primary chemotherapy treatment, rather than surgery, is recommended for NF-1-associated, asymptomatic and mild symptomatic OPG patients. The benefits of decompression must be weighed against the risks and complications of the surgery. Techniques, such as diffusion tensor tractography and intraoperative visual evoked potentials (VEP) monitoring, rationalize surgical planning and reduce the risks of surgery (33, 34).

Other studies described that young age, sporadic type, poor IVA, optic disk pallor, tumor volume, and tumor involvement of the chiasma/hypothalamus/post-chiasm may be associated with poor VA prognosis (17, 35–39). One patient with tumor recurrence suffered significant further visual impairment in the present study, which suggests that recurrence may be associated with vision deterioration. However, due to the small number of recurrence cases in the present study, we did not perform statistical analysis. After decompression surgery treatment, three-fifths of the eyes maintained normal vision at all times, and one-third of vision-impaired eyes had VA improvement. One study reported VA improvement of ~18.18% in sporadic OPG after chemotherapy (38), whereas ours yielded a greater improvement rate of 29.87% after decompression surgery. This may suggest that some children may benefit from better VA outcome from decompression surgery as compared to chemotherapy. We also found a positive correlation between the vision level of IVA and VA improvement (*G* = 0.686, *p* < 0.001). Multivariate analysis revealed a correlation between VA improvement and IVA (OR, 1.987; 95% CI, 1.249–3.162; *p* = 0.004) and large-size tumors (OR, 0.205; 95% CI, 0.125–2.073; *p* = 0.048), and there was only a marginal correlation between VA outcome and the ICH (*p* = 0.053). Decompression surgery worked better in patients with milder VA impairment and had a limited effect on VA improvement in patients with large-size tumors. OPG is generally considered a slow-growing tumor, and some researchers divide the tumor into an infiltrative endophytic type and inflated type based on diffusion tensor tractography (DTT) images (33). The different manifestations on DTT suggest that the tumor damages the optic nerve in several different ways, including invasion and compression. Relief of compression may help the optic nerve partially regain function, but the detailed mechanism must be further elucidated (40). Long-term chronic compression caused by large tumors may limit the ability to restore vision (41). The post-operative VA improvement rate of small-size tumor was also lower than that of medium-size tumors, but the difference was not statistically significant (OR, 0.509; 95% CI, 0.042–0.987; *p* = 0.346). All of the patients with post-surgical blindness were in the small-size tumor group. Therefore, surgery for small-volume tumors should be performed cautiously. The decompression must be performed within appropriate conditions to minimize the surgical risks and maximize its benefits. ROC analysis found that the AUC of IVA + tumor size predicting VA improvement was 0.831, and the cutoff points for IVA level and tumor volume were 4.5 and 43.50 cm^3^, respectively. Eyes with IVA ≥ level 5 tended to have better VA improvement rates after surgical treatment, and the vision recovery probability for eyes with tumor volume ≥43.50 cm^3^ was much lower.

## Limitation

This study was a retrospective article with several limitations. The patients included in this study did not share a regular interval of assessment of post-surgical vision, and the duration from IVA to LVA assessment varied widely. Most patients in this study received AT treatment after surgery, and the effects of surgery and adjuvant therapy on VA could not be isolated. However, these limitations are likely inherent to retrospective studies. The results in this study could not reflect the fluctuations of VA during the treatment, but it reflected the overall VA outcome after the entire treatment of sporadic CHG patients who received surgery. Young children were excluded because they could not cooperate with the VA test. Therefore, the inclusion of subjects was biased. The application of VEP may help evaluate the eyesight of young children, and regular assessment of the patient's VA to obtain continuous data will help evaluate the efficacy of each stage of treatment. Prospective multicenter clinical trials that meet uniform vision assessment standards are needed in the future.

## Conclusions

The visual prognosis of some sporadic CHG patients may benefit from decompression surgery. IVA and tumor size correlated with VA improvement after primary intratumor partial resection. Better IVA predicted a higher vision improvement rate, and children with IVA ≥ level 5 were more likely to achieve visual improvement after decompression surgery. Decompression had limited effectiveness on vision improvement in patients with tumor volume ≥ 43.50 cm^3^. Performing resections under an extent of 50% was safe and did not reduce the effect of improving VA. Because some children develop complications that affect QOL, the benefits of decompression surgery must be balanced against the risks. The indication for surgery should be estimated strictly, and an interdisciplinary approach is required to assess the optimal treatment strategy for each OPG child.

## Data Availability Statement

The data analyzed in this study is subject to the following licenses/restrictions: The processed data required to reproduce these findings cannot be shared at this time as the data also forms part of an ongoing study. Requests to access these datasets should be directed to Yongji Tian, ttyysw1tyj@163.com.

## Ethics Statement

The studies involving human participants were reviewed and approved by Ethics Review Committee of the Beijing Tiantan Hospital. Written informed consent from the participants' legal guardian/next of kin was not required to participate in this study in accordance with the national legislation and the institutional requirements.

## Author Contributions

CLia, HZ, and ZH: acquisition of data. CLia and ZL: analysis and interpretation of data and statistical analysis. WL, CLi, JG, and ZM: critically revising the article. YT: funding acquisition and study supervision. CLia and YT: conception and design. All authors contributed to the article and approved the submitted version.

## Conflict of Interest

The authors declare that the research was conducted in the absence of any commercial or financial relationships that could be construed as a potential conflict of interest.
