# Corrigendum: The Visual Acuity Outcome and Relevant Factors Affecting Visual Improvement in Pediatric Sporadic Chiasmatic–Hypothalamic Glioma Patients Who Received Surgery

**DOI:** 10.3389/fneur.2022.914268

**Published:** 2022-05-17

**Authors:** Chihyi Liao, Heng Zhang, Zhiming Liu, Zhe Han, Chunde Li, Jian Gong, Wei Liu, Zhenyu Ma, Yongji Tian

**Affiliations:** ^1^Department of Neurosurgery, Beijing Tiantan Hospital, Capital Medical University, Beijing, China; ^2^Beijing Key Laboratory of Brain Tumor, China National Clinical Research Center for Neurological Diseases, Center of Brain Tumor, Beijing Institute for Brain Disorders, Beijing, China

**Keywords:** chiasmatic–hypothalamic glioma, optic pathway glioma, visual acuity, prognostic factors, primary surgical treatment

In the original article, there was an error. The AUC of IVA + tumor size predicting VA improvement was incorrectly stated. A correction has been made to Results, Paragraph 6:

In the ROC analysis, the AUC of IVA predicting VA improvement was 0.787 (95% CI: 0.674–0.900, *p* < 0.001), and the AUC of IVA + tumor size predicting VA improvement was 0.831 (95% CI: 0.729–0.933, *p* < 0.001). For medium to large tumors, the AUC of tumor volume predicting VA outcome was 0.748 (95% CI: 0.641–0.883, *p* =0.005). The cutoff point of IVA level was 4.5 (sensitivity = 73.91%, specificity = 75.93%, positive predictive value = 56.67%, negative predictive value = 87.23%, accuracy=75.32). The cutoff point of tumor volume was 43.50 cm^3^ (sensitivity = 95.65%, specificity = 31.48%, positive predictive value = 37.29%, negative predictive value =94.44%, accuracy =50.65%) (**Table 4**).

Additionally, in the original article there was an error in the vision levels reported. A correction has been made to Methods, Paragraph 2:

According to the range of vision loss reported by the International Council of Ophthalmology (12), we converted the categories of vision impairment to a seven-level scale: level 7 to level 1 represent normal vision (≥0.8), mild visual impairment (0.32–0.63), moderate visual impairment (0.125–0.25), severe visual impairment (0.05–0.1), profound visual impairment (0.02–0.04), near-blindness (<0.02), and light perception or blindness, respectively. The VA outcome of patients was categorized into “VA improvement” and “no VA improvement.” If the LVA level was higher than the IVA level, the outcome was defined as “VA improvement.” If not, the outcome was defined as “no VA improvement.” “Maintained” was used when the LVA level was equal to the IVA level, and “deteriorated” was used when the LVA level was lower than the IVA level. Tumor volume was calculated using the ellipsoid volume formula “A × B × C × π/6” (**Figure 1**) (13). Tumors were divided into small, medium, and large size groups by tertiles. Tumor volume ≤13.80 cm^3^ was categorized as a small-size tumor, 13.80 cm^3^ < tumor volume ≤34.60 cm^3^ was categorized as a medium-size tumor, and the tumor volume > 34.60 cm^3^ was categorized as a large-size tumor.

The authors apologize for this error and state that this does not change the scientific conclusions of the article in any way. The original article has been updated.

## Publisher's Note

All claims expressed in this article are solely those of the authors and do not necessarily represent those of their affiliated organizations, or those of the publisher, the editors and the reviewers. Any product that may be evaluated in this article, or claim that may be made by its manufacturer, is not guaranteed or endorsed by the publisher.

