# Supplementary material for: The Visual Acuity Outcome and Relevant Factors Affecting Visual Improvement in Pediatric Sporadic Chiasmatic–Hypothalamic Glioma Patients Who Received Surgery
Source: Front Neurol. 2020 Aug 19;11:766. doi: 10.3389/fneur.2020.00766 (PMC7466562; doi:10.3389/fneur.2020.00766)
Supplement: Supplementary file 1 [file Data_Sheet_1.docx]

Supplementary Material

# Supplementary Tables

Table S1 Vision level of IVA and LVA for 90 eyes of 45 sporadic CHG children

| LVA | IVA | | | | | | | Total |
| --- | --- | --- | --- | --- | --- | --- | --- | --- |
|  | Level-7 | Level-6 | Level-5 | Level-4 | Level-3 | Level-2 | Level-1 |  |
| Level-7 | 8 | 15 | 1 | 1 |  |  |  | 25 |
| Level-6 | 4 | 4 | 1 | 1 |  |  |  | 10 |
| Level-5 |  | 2 | 2 |  |  | 1 |  | 5 |
| Level-4 |  | 2 |  | 6 | 2 |  |  | 10 |
| Level-3 |  |  |  | 1 | 6 |  | 1 | 8 |
| Level-2 | 1 |  | 1 | 1 | 2 | 6 |  | 11 |
| Level-1 |  | 1 | 1 | 1 | 2 | 5 | 11 | 21 |
| Total | 13 | 24 | 6 | 11 | 12 | 12 | 12 | 90 |

Abbreviations: IVA, initial visual acuity; LVA, last visual acuity

Table S2 Correlation of postsurgical complications with disease characteristics and treatment

| Variable | *p* Value | | | | | |
| --- | --- | --- | --- | --- | --- | --- |
|  | Complications affecting QOL | Endocrine disorder | Infection | Diabetes insipidus | Electrolyte disorder | Subdural effusion |
| Sex | 0.350 | 0.744 | 0.658 | 0.731 | 0.493 | 0.137 |
| Age | 0.378 | 0.213 | 0.686 | 0.121 | 0.736 | 0.501 |
| ICH | >0.999 | 0.072 | 0.027* | 0.592 | 0.500 | 0.495 |
| Shunt | >0.999 | 0.725 | 0.650 | 0.502 | 0.699 | 0.067 |
| Tumor size | 0.594 | 0.174 | 0.858 | 0.310 | 0.048* | >0.999 |
| Surgical approach | >0.999 | 0.279 | 0.149 | >0.999 | 0.091 | 0.364 |
| Resection extent | 0.042* | 0.917 | 0.863 | 0.512 | 0.666 | 0.733 |

**p* value < 0.05

TABLE S3 Correlation of disease characteristics with VA improvement in the vision-impaired eyes (per eye)

| Variable |  | VA improvement  n=23 | No VA improvement n=54 | *p* |
| --- | --- | --- | --- | --- |
| Sex | Male | 15 | 34 | 0.851 |
|  | Female | 8 | 20 |  |
| Age | ≤5 years | 13 | 23 | 0.262 |
|  | >5 years | 10 | 31 |  |
| Vision impaired type | Unilateral | 2 | 9 | 0.576 |
|  | Bilateral | 21 | 45 |  |
| ICH | Yes | 16 | 26 | 0.084 |
|  | No | 7 | 28 |  |
| Other ophthalmic symptome | Yes | 4 | 10 | >0.999 |
|  | No | 19 | 44 |  |
| Precocious puberty | Yes | 4 | 9 | >0.999 |
|  | No | 19 | 45 |  |
| IVA | Level-6 | 15 | 9 | 0.002 |
|  | Level-5 | 2 | 4 |  |
|  | Level-4 | 2 | 9 |  |
|  | Level-3 | 2 | 10 |  |
|  | Level-2 | 1 | 11 |  |
|  | Level-1 | 1 | 11 |  |
| AT | RT | 17 | 35 | 0.913 |
|  | CT | 1 | 4 |  |
|  | RT+ Oral temozolomide | 4 | 10 |  |
|  | Observation | 1 | 5 |  |
| Pathology | PA | 6 | 18 | 0.791 |
|  | PXA or PA/PXA | 12 | 23 |  |
|  | Diffuse astrocytoma | 3 | 10 |  |
|  | Ganglioglioma | 2 | 2 |  |
|  | Anaplastic astrocytoma | 0 | 1 |  |
| Shunt | Yes | 8 | 11 | 0.179 |
|  | No | 15 | 43 |  |
| Surgical approach | Longitudinal fissure | 12 | 24 | 0.149 |
|  | Subtemporal | 0 | 8 |  |
|  | Transcallosal interforniceal | 11 | 22 |  |
| Tumor size | Small | 7 | 19 | 0.020 |
|  | Medium | 13 | 14 |  |
|  | Large | 3 | 21 |  |
| Resection extent | >70% | 13 | 25 | 0.592 |
|  | 50-70% | 6 | 14 |  |
|  | <50% | 4 | 15 |  |

Abbreviations: IVA, initial visual acuity; LVA, last visual acuity; ICH, intracranial hypertension; AT, adjuvant treatment; RT, radiotherapy; CT, chemotherapy; PA, pilocytic astrocytoma; PMXA,pilomyxoid astrocytoma.
